# Detection of soluble solids content in tomatoes using full transmission Vis-NIR spectroscopy and combinatorial algorithms

**DOI:** 10.3389/fpls.2024.1500819

**Published:** 2024-11-11

**Authors:** Letian Cai, Yizhi Zhang, Zhonglei Cai, Ruiyao Shi, Sheng Li, Jiangbo Li

**Affiliations:** ^1^ Intelligent Equipment Research Center, Beijing Academy of Agriculture and Forestry Sciences, Beijing, China; ^2^ National Agricultural Intelligent Equipment Engineering Technology Research Center, Beijing, China

**Keywords:** tomato, online detection, feature selection, internal quality assessment, soluble solids content

## Abstract

**Introduction:**

Soluble solids content (SSC) is an important indicator for evaluating tomato flavor, and general physical and chemical methods are time-consuming and destructive.

**Methods:**

This study utilized full transmittance visible and near infrared (Vis-NIR) spectroscopy for multi-posed data acquisition of tomatoes in different orientations. The role of two directions (Z1 and Z2) and four preprocessing techniques, as well as three wavelength selection methods in the exploitation of SSC regression models was investigated.

**Results:**

After using the Outlier elimination method, the spectra acquired in the Z2 direction and the raw spectral data processed by preprocessing methods gave the best result by the PLSR model (*R_p_* = 0.877, *RMSEP* = 0.417 %). Compared to the model built using the full 2048 spectral wavelengths, the prediction accuracy using 20 wavelengths obtained by a combination wavelength selection: backward variable selection - partial least squares and simulated annealing (BVS-PLS and SA) was further improved (*R_p_* = 0.912, *RMSEP* = 0.354 %).

**Discussion:**

The findings of this research demonstrate the efficacy of full-transmission visible-near infrared (Vis-NIR) spectroscopy in forecasting SSC of tomatoes, and most importantly, the combination of the packing method in wavelength selection with an intelligent optimization algorithm provides a viable idea for accurately and rapidly assessing the SSC of tomatoes.

## 1 Introduction

Tomatoes, known as the fruit of vegetables, are widely grown around the world, and their consumption helps to consume fiber, antioxidants and a variety of minerals, which can reduce the likelihood of developing cancer and chronic illnesses, so tomatoes are popular with the public (Ozturkoglu-Budak and Aksahin, 2016). With the increase of tomato demand, the quality of tomato has been paid more and more attention. High quality fresh tomatoes require high nutritional value and good taste. Soluble solid content (SSC) refers to the percentage of soluble substances such as soluble sugar and organic acid in tomatoes, which is a very important indicator to measure the internal quality of tomatoes and is closely associated with consumers’ perceptions of the intrinsic quality characteristics of the fruit (Zheng et al., 2024). The conventional approach for measuring the SSC of tomatoes typically employs the refractometer technique, which necessitates the extraction of juice from the fruit followed by titration. This procedure is not only time-intensive but also destructive, rendering it impractical for large-scale fruit analysis (Tan et al., 2022). Consequently, the development of a non-destructive and efficient measurement technique for the quality assessment of tomatoes is of considerable importance.

Spectral analysis technology is usually used to study the relationship between light and matter, and obtain spectral information through the response of matter to light, so as to reflect the physical or chemical information of the target region. Visible near-infrared (Vis-NIR) spectroscopy analysis is one of the mainstream methods for non-destructive examination of fruit internal quality (Mei and Li, 2023). Zhang et al. (2021) achieved accurate prediction of tomato SSC by setting different Vis-NIR spectral ranges. Brito et al. (2021) collected the NIR spectral data of tomato and established a partial least squares (PLS) regression model for the detection of SSC of tomato by using orthogonal signal correction, and the standard deviations of the calibration and validation sets were obtained to be 0.52% and 0.56%, respectively. Huang et al. (2018) used a self-constructed system for spatially resolved spectroscopy to detect tomato quality, and the results proved that the system had a significant advantage over the traditional single-point Vis/NIRS instrument in tomato SSC assessment, with *Rp* and *RMSEP* of 0.801 and 0.38%, respectively. For the characteristics of the heterogeneous structure of tomato, it is necessary to obtain as much information as possible about the interior of the tomato, however, typical Vis-NIR spectroscopy detection technique is flawed because single-point measurements are capable of providing only a restricted amount of spatial information regarding the sample. Yang et al. (2022) optimized the optical path, light intensity and other detection settings of the Vis-NIR diffuse transmittance system and developed a compensation model based on the physiological traits of tomato to obtain a favorable accuracy of SSC detection in tomato (Rp = 0.91, PMSEP = 0.17%). In contrast to the reflection mode, the transmission mode is capable of providing a greater amount of information regarding the internal structure and material of the tomato fruit; in addition, the implementation of full transmission and continuous data acquisition methodologies can address the constraints associated with the conventional single-point Vis-NIR measurement technique, thereby facilitating a thorough characterization of the entire tomato’s properties.

Owing to advancements in contemporary analytical methodologies, Vis-NIR spectroscopy makes it easy to measure information about objects characterized by a large number of spectral bands in a short period of time. Pluralistic metrological techniques were used to extract the most useful information from redundant data, and enhanced quantitative calibration models were developed through the systematic evaluation of characteristic wavelengths or wavelength intervals through variable selection methods. Numerous studies in the fields of statistics and data analysis elucidate various methodologies for the selection of variables which can be broadly categorized into three groups: filtering, wrapping and embedding. Filtering method selects variables and evaluates them independently by introducing thresholds (e.g., load weights or regression coefficients). Liu et al. (2008) used NIR spectroscopy to differentiate fruit vinegar varieties based on a filtering method. Inoue et al. (2012) used a correlation coefficient filtering method to accurately assess the nitrogen content of rice canopies. The wrapping method takes into account the correlation between the variables and selects them by evaluating the impact of the combination of variables on the model performance. Cai et al. (2008) showed that the uninformative variable elimination (UVE) - PLS wrapping method could more accurately predict nicotine content in tobacco samples. The embedding method involves the selection of variables while building the model, and the interplay between variable selection and sample categorization leads to a reduction in the time required for analysis. Ning et al. (2022) used the least absolute shrinkage and selection operator (LASSO) embedding method to select variables from preprocessed NIR spectra, and realized the quantitative detection of mycotoxins in wheat kernels by develop a SVM model. The above analysis indicated that the amount of data can be greatly compressed by using different variable selection and its combination algorithms, thus the operational efficiency of the model was enhanced; concurrently, the predictive accuracy and stability of the model were further augmented through the removal of nonlinear or extraneous variables.

In conclusion, the primary objective of this research was to develop an optimal model for the detection of SSC in tomatoes utilizing full transmission Vis-NIR spectroscopy. The specific aims of the study were as follows: (1) To evaluate the effect of different tomato placement orientations on spectral prediction accuracy; (2) To investigate the effect of different spectral pretreatment methods on tomato transmission spectrum; (3) To screen the spectral wavelengths using different feature selection algorithms, and determine the most effective predictive model by evaluating both its accuracy and the time required for modeling.

## 2 Materials and methods

### 2.1 Sample preparation

Ninety samples of fresh tomatoes (‘Provence’ variety) were purchased from a vegetable supermarket in Beijing, China. ‘Provence’ tomatoes have thin skins, juicy flesh, and a rich reddish color when ripe, and the used samples were free of any surface damage. To mitigate temperature variations that may lead to inaccuracies in measurements, the tomatoes were maintained at a temperature of 20°C and a relative humidity of 60% for a duration of 24 hours prior to the collection of spectral and SSC data. During the development of the model, the ratio of sample numbers between the calibration set and the prediction set was established at 3:1.

### 2.2 Online full transmission spectrum acquisition system

**Figure 1:**
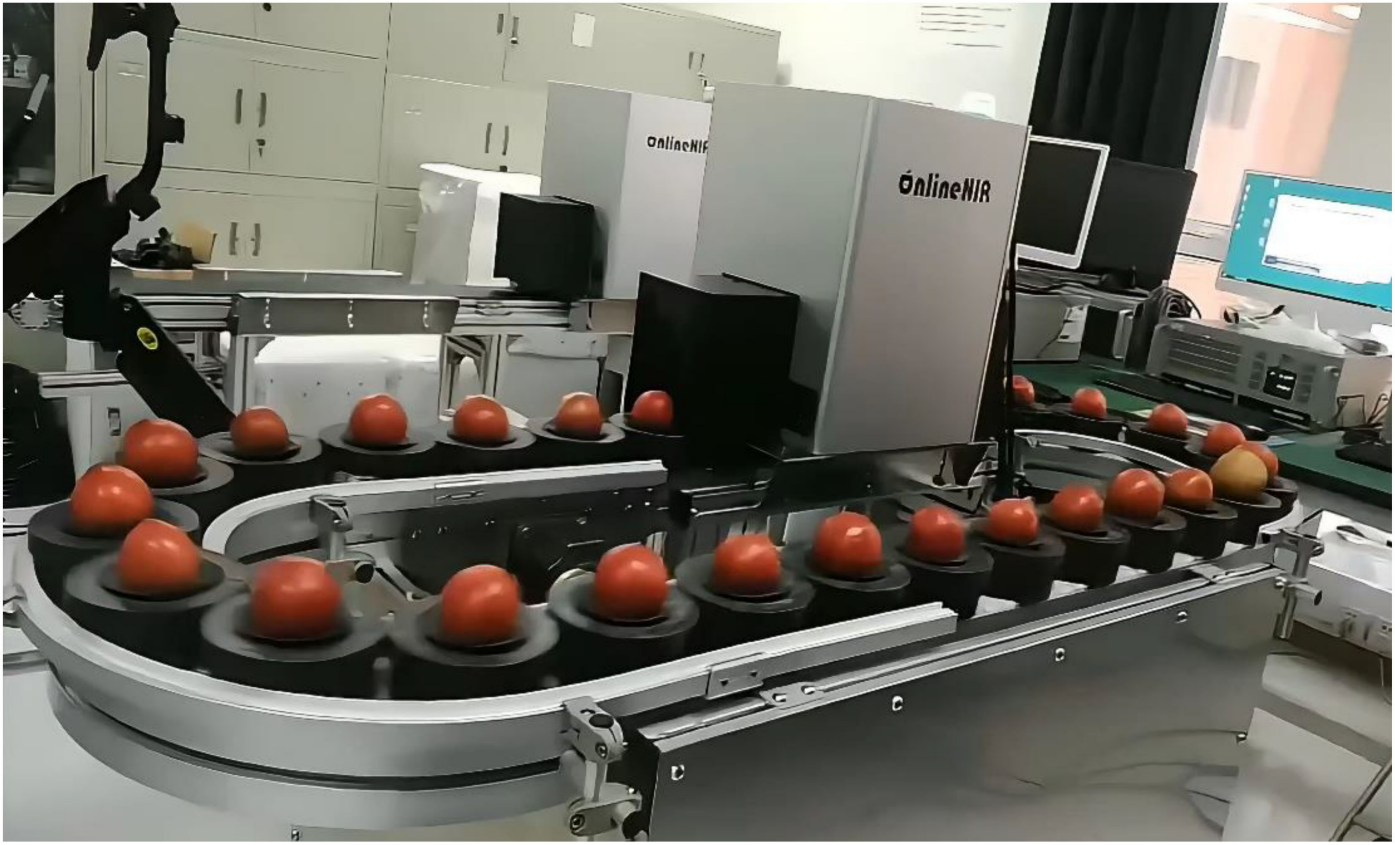
Full transmission Vis-NIR online tomato detection system.

The spectral data of the tomato samples were collected by using a full transmission Vis-NIR online detection system shown in **Figure 1**, and the main units include: a highly sensitive spectrometer (wavelength range: 560-1072 nm, wavelength interval: 0.25 nm, integration time: 5 ms), a speed-adjustable moving platform, a dark box, relative position sensors, an illumination unit consisting of a 150 W halogen lamp with focusing and attenuating device and a computer for the control system. The illumination unit and the spectrometer are placed 150 mm apart on each side of the conveyor belt in the dark box.

**Figure 2:**
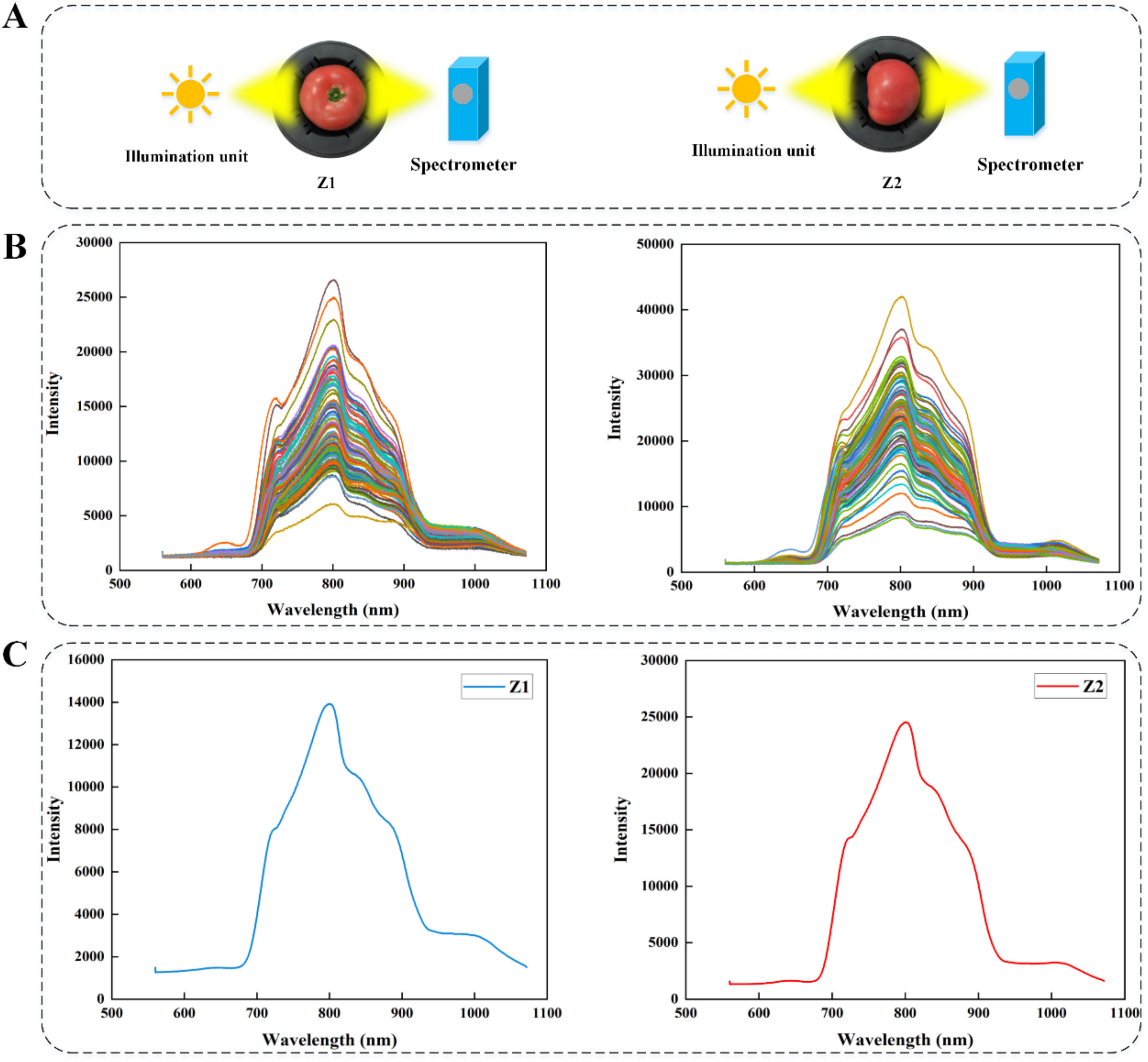
**(A)** Tomato detection orientations. **(B)** Multi-point raw spectral curves. **(C)** Spectral curve after averaging of multi-point raw data.

In order to assess the effect of the complex cavity inside the tomato on the accuracy of the on-line detection of SSC, each sample passed through the measuring device on a conveyor belt in two different orientations, Z1 orientation: the stem-calyx axis of the test samples were perpendicular to the conveyor belt, and the stems were facing upward; Z2 orientation: the stem-calyx axis of the test samples were parallel to the conveyor belt, and the stems were facing toward the spectrometer, and the orientation of the test samples were shown schematically in **Figure 2A**.

### 2.3 SSC measurement

SSC refers to the percentage of solvable content in the tomato juice, which is mainly composed of nutrients such as soluble sugar (C₆H₁₂O₆) and organic acids (-COOH), and is an important indicator of tomato quality and fruit processing characteristics. After online spectrum acquisition, the traditional destructive method was used to measure the SSC of tomatoes immediately. The measuring instrument was digital Abbe refractometer (model PAL-1, Atago Co., Tokyo, Japan). The intact tomatoes were first chopped and pressed in a wall breaker to make tomato juice, then the tomato juice was filtered through gauze and the juice was dripped into a beaker. After sufficient shaking, about 0.3 ml of juice was dripped onto a digital refractometer (zero-corrected) and the SSC value was recorded manually.

### 2.4 Data preprocessing

At the time when the tomato samples just reached the detection position and left the detection position, the light signal from the spectrometer passed through only a small portion of the sample’s pericarp tissue. Due to the high optical intensity, these portions of the spectrum should be removed prior to modeling. The extent of removal in this study was about halfway between the two sides of the sample pericarp, which is close to the ideal range. The concluding spectral data obtained encompassed the majority of the pulp and cavity regions within each tomato sample, representing the primary internal quality areas of the entire tomato.

To further improve the spectral data quality, the pre-processing algorithms were generally used to reduce the noise and interference of the instrument and background before building the prediction model. Gaussian filter (GF) is a linear smoothing filter based on the Gaussian function (Zhang et al., 2024), The processing process is as follows: Firstly, the filter parameters are determined, then the GF is constructed, the weight of each data point in the filter is calculated and normalized according to the window size and standard deviation, and finally the data is convolved. Due to the influence of instrument accuracy and background noise, the spectral signals obtained by the spectrometer contain both useful information and random errors superimposed on them at the same time, and the use of smoothing algorithms reduces the noise and improves the signal-to-noise ratio (Chen et al., 2014). Standard normal variate (SNV) is mainly used to eliminate the effects of surface scattering as well as optical range changes (Dhanoa et al., 2023). The processing process is as follows: Firstly, for a given set of spectral data, the mean and standard deviation of all wavelength points in the spectrum are calculated; the data value for each wavelength point is then subtracted from the mean of the sample spectrum and divided by the standard deviation. Multiplicative scatter correction (MSC) is primarily employed to mitigate the scattering effects arising from heterogeneous particle distribution and variations in particle size (Li et al., 2018). The processing process is as follows: Firstly, a spectrum considered representative is selected as the reference spectrum. Then, for each spectrum to be processed, the linear relationship between it and the reference spectrum is calculated. Finally, the data of each wavelength point of the spectrum are corrected by using the obtained linear equation parameters. In this study, GF, SNV and SMC preprocessing algorithms were used to refine the tomato full transmission spectrum data.

### 2.5 Prediction model and evaluation indicators

Partial least squares regression (PLSR) is a multivariate factorial regression technique frequently employed in the field of spectral analysis. PLSR constructs predictive models by finding the optimal linear combinations of independent and dependent variables and extracting the latent variables (*LVs*) that maximize the correlation between input and output variables (Diniz et al., 2015). In addition, PLSR demonstrates computational efficiency, particularly in scenarios where the sample size is limited while the number of variables is extensive (Li et al., 2023). In this research, a PLSR model was developed to elucidate the quantitative relationship between the spectral matrix of tomatoes (X) and the matrix of SSC values (Y). The root mean square error of cross-validation (*RMSECV*) was employed to ascertain the optimal number of *LVs*.

Calibration correlation coefficient ( $R_{c}$ ), root mean square error of calibration (*RMSEC*), prediction correlation coefficient ( $R_{p}$ ), and root mean square error of prediction (*RMSEP*) were used to assess model performance. See Equation 1 for specific calculations. Typically, models with higher correlation coefficients ( $R_{c}$ and $R_{p}$ ) and lower root-mean-square errors (*RMSEC*, *RMSECV*, and *RMSEP*) can be considered to meet expectations. Matlab 2023b (Mathworks, Natick, MA) performed the development of all model programs.


(1) $$\begin{array}{c} R_{c},R_{p}=\sqrt{1-\frac{\sum_{i=1}^{n}{\left(y_{i}-\hat{y_{i}}\right)}^{2}}{\sum_{i=1}^{n}{\left(y_{i}-\bar{y_{i}}\right)}^{2}}}\;;\;\;\;\\ RMSEC,RMSEP=\sqrt{\frac{1}{n}\sum_{i=1}^{n}{\left(y_{i}-\hat{y_{i}}\right)}^{2}} \end{array}$$

where $y_{i\;}$ , $\hat{y_{i}}$ and $\bar{y_{i}}$ denote the measured, predicted and mean values of the $i_{th}$ tomato sample in the calibration or prediction set, respectively, and n represents the total quantity of tomato samples included in either the calibration or prediction dataset.

### 2.6 Wavelength selection methods

The full spectrum contains 2048 wavelengths with a large number of uncorrelated and co-linear variables. Moreover, this adds complexity to the model, a large number of wavelengths may introduce interference, which can make the model run slower and less accurate (Luo et al., 2022). Therefore, it is important to find a variable selection algorithm to simplify the spectral data without reducing the accuracy of the model (Li et al., 2024).

#### 2.6.1 Backward variable selection PLS

BVS-PLS is a packaging technique that utilizes the PLS regression algorithm and operates iteratively through a process of backward selection (Fernández et al., 2009). In this study, *RMSECV* was used as a criterion for variable optimization, which was calculated as follows:

Step-1: The w full-spectrum wavelengths were divided into m groups, each containing n number of wavelengths, w=m×n.

Step-2: The PLS regression model was fitted to a grouped dataset containing all n wavelengths and *RMSECV* was calculated as the initial value for the iteration.

Step-3: Cycled through the different groups. Deleted one wavelength group at a time and fit the PLS model to the remaining m−1  wavelength groups, with the wavelength group with the largest decrease in *RMSECV* being discarded.

Step-4: Step-3 was repeated, iteratively discarding wavelength sets until a point was reached where the *RMSECV* was no longer decreasing. At this point all wavelengths with poor correlation with the tomato SSC to be predicted have been eliminated.

During the algorithmic loop, the data was tested using leave-one-out cross-validation.

#### 2.6.2 Simulated annealing algorithm

Simulated annealing algorithm is a widely used heuristic stochastic intelligent optimization algorithm (Ekinci et al., 2023), SA distinguishes itself from other optimization algorithms in that it can receive solutions that are worse than the previous result (Suman and Kumar, 2006; Lin et al., 2018), and thus is able to obtain a more varied solution space that is not prone to falling into local optima (Shi et al., 2024). The specific calculation steps are as follows:

Step-1: Initialized the annealing table, which consisted of the initial temperature $T_{0}$ , the cooling parameter α in the temperature update function, the maximum number of iterations L , and the termination temperature $T_{e}$ .

Step-2: Randomly generated a $\;h_{0}$ as the current solution $h_{k}$ .

Step-3: Generated a neighborhood solution $h^{\prime }$ . The selection rule of the neighborhood solution was: if the objective function was continuous, generated a random vector $z_{k}$ ; if the objective function was discrete, generated a random offset $z_{m}$ , and obtained a neighborhood solution by Equation 2:


(2) $$\begin{array}{c} h^{\prime }=\{\begin{array}{c} h_{k}+z_{k},\;\;continuous\;function\\ h_{\left(k+m\right)},\;\;\;\;\;\;discrete\;function \end{array} \end{array}$$

Step-4: Calculated and compared the fitness functions $C\left(h_{k}\right)$ and $C\left(h^{\prime }\right)$ , if $C\left(h^{\prime }\right)<C\left(h_{k}\right)$ , received $h^{\prime }$ ; if $C\left(h^{\prime }\right)>C\left(h_{k}\right)$ , extracted a random number from a uniform distribution in [0,1] that was less than the probability value and accepted the change $h^{\prime }$ .

Step-5: Determined whether the algorithm reaches the maximum number of iterations L , if it meets, then go to step-6, if not, then return to step-3.

Step-6: Ascertain whether the termination criterion has been met; if it has been met, present the optimal solution; if it was not satisfied, update the temperature using the temperature update function T(k+1)=α×T(k) and return to step-3.

## 3 Results and discussion

### 3.1 Influence of spectral acquisition orientations on the prediction of tomato SSC

Tomato is usually not a completely uniform structure. There may be differences in the internal organization of different parts of the tomato, such as the cell structure, water distribution, sugar content, etc. near the stem and in the middle of the tomato. By studying different spectral acquisition locations, the characteristics of different regions inside the tomato can be more comprehensively understood, so as to find the most suitable spectral acquisition strategy for specific tomato fruit varieties, and improve the universality and accuracy of the prediction model. **Figure 2B** showed the original spectral data collected by multi-point full transmission measurement way. It can be seen that the intensity of each spectral curve was relatively large. At the same time, due to the different acquisition locations and the variability of physical and chemical properties of tomatoes, there were obvious intensity differences between different spectral curves. **Figure 2C** showed the spectral curve after averaging the original data from multiple points. Generally speaking, Z1 orientation: the stem-calyx axis of the test samples were perpendicular to the conveyor belt, and Z2 orientation: the stem-calyx axis of the test samples were parallel to the conveyor belt, the trend and characteristics of the spectral curves obtained by the two acquisition methods were basically similar, but the intensity of the optical signal collected in the orientation of Z2 was higher than that of Z1, this phenomenon can be attributed to the impact of the internal cavity architecture of the tomato sample on the trajectory of light propagation. Due to the shorter optical path distance in the Z2 direction, which was less affected by the structure of the tomato cavity, the transmission spectral signals obtained were stronger.

For the raw transmission spectral data collected in different orientations, the corresponding PLSR models were established to evaluate their prediction performance. As can be seen from **Table 1**, for Z1 orientation, $R_{c}$ and *RMSEC* of model were 0.951 and 0.258%, respectively, $R_{p}$ and *RMSEP* were 0.637 and 0.713%, respectively; and for Z2 orientation, $R_{c}$ and *RMSEC* of model were 0.917 and 0.338%, respectively, $\;R_{p}$ and *RMSEP* were 0.757 and 0.583%, respectively. Compared with Z1, the model built in the Z2 orientation improved the accuracy of the prediction set while avoiding potential overfitting of the correction set. For the two detection orientations, the performance of the model was also improved with the increase of spectral intensity. Based on the above analysis, the raw spectral data obtained in the Z2 orientation were used for the subsequent modeling steps.

**Table 1:** SSC of tomatoes was predicted by full spectrum PLS model in different acquisition orientations.

Acquisition orientation	*LVs*	$R_{c}$	*RMSEC (%)*	$R_{p}$	*RMSEP (%)*
Z1	11	0.951	0.258	0.637	0.713
Z2	10	0.917	0.338	0.757	0.583

Z1: the stem-calyx axis of the test samples was perpendicular to the conveyor belt, Z2: the stem-calyx axis of the test samples was parallel to the conveyor belt.

### 3.2 Spectrum preprocessing and abnormal sample removal

**Figure 3:**
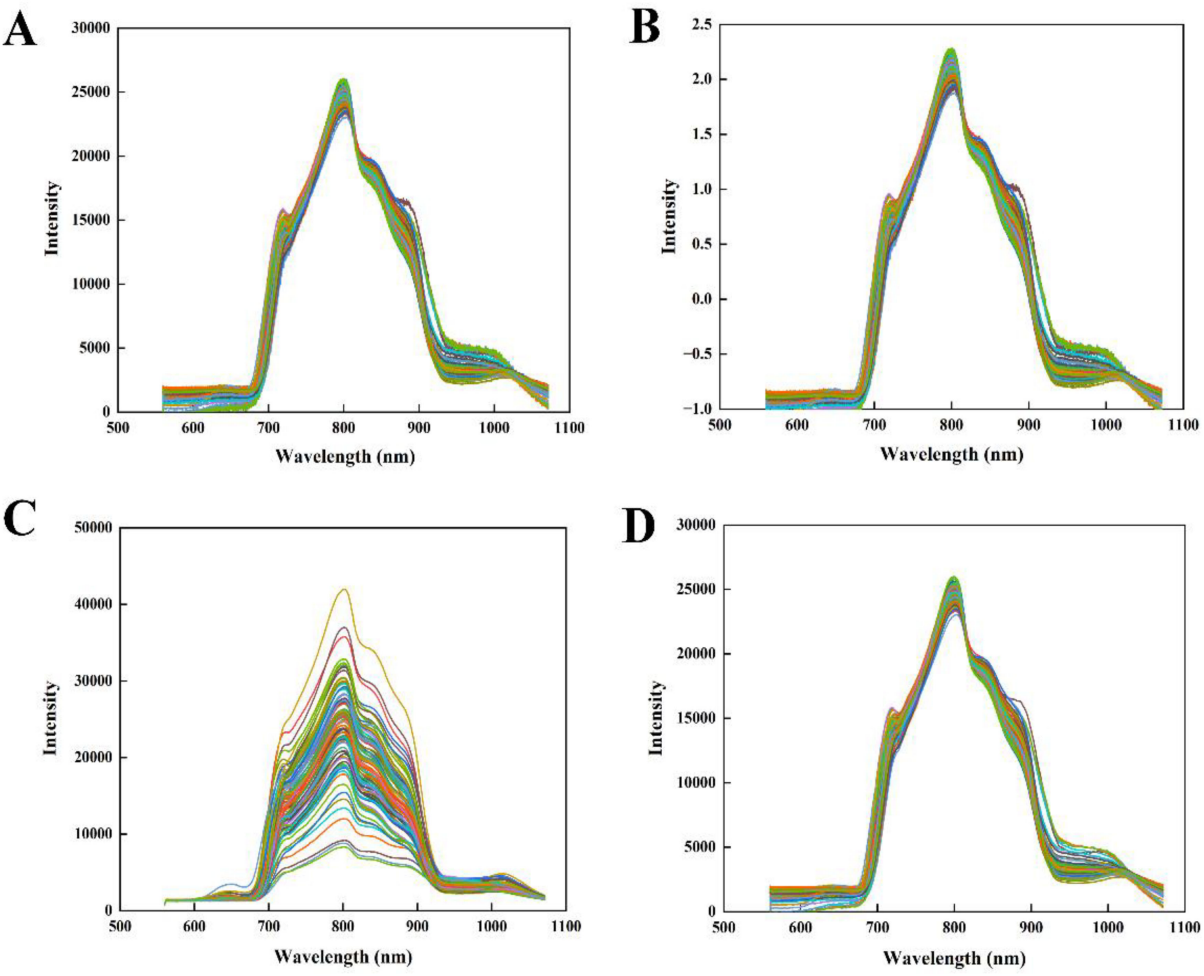
Spectral preprocessing of all samples.**(A)** MSC, **(B)** SNV, **(C)** GF, and **(D)** GF and MSC.

The spectrum obtained by the instrument encompasses not only the chemical characteristics of the sample but also extraneous information and noise, including electrical interference, background signals from the sample, and stray light. In this study, four preprocessing methods, including MSC, SNV, GF and GF and MSC, were used to process the original transmission spectrum of the tomato samples, and the spectral curves after preprocessing were shown in **Figure 3**. It can be seen that the MSC and SNV algorithms reduced the influence of the heterogeneous particle distribution in tomato. Compared with the original spectra in **Figure 2B**, the spectral curves after GF treatment reduced the undesirable noise and presented smooth curves in the figure. The spectral curves after GF and MSC treatment also showed the same spectral trend as in the above analysis.

The results in **Table 2** showed that the $R_{c}$ was improved after the pre-processing of MSC and SNV, and the *RMSEC* showed a significant decrease, which may be the result of suppressing the surface scattering of the samples. However, the improvement of the model performance in the prediction set was limited. After the GF treatment, although the performance of the model on the calibration set appeared to be degraded, the accuracy of the model on the prediction set was significantly improved. As a comparison, after GF and MSC combination preprocessing, model obtained the better performance ( $R_{c}\;$ = 0.927, *RMSEC* = 0.316%, $R_{p}$ = 0.852, *RMSEP* = 0.456%). Obviously, the GF and MSC preprocessing process amplified the spectral properties and resulted in clearer and more consistent spectra, which in turn improved the stability of the data.

**Table 2:** Prediction results of tomato SSC by PLSR model with preprocessed full wavelengths.

Number of samples	Methods	*LVs*	$R_{c}$	*RMSEC (%)*	$R_{p}$	*RMSEP (%)*
96 samples	RAW	10	0.917	0.338	0.757	0.583
	MSC	11	0.976	0.184	0.786	0.547
	SNV	12	0.974	0.189	0.784	0.550
	GF	12	0.845	0.461	0.831	0.487
	GF+MSC	12	0.927	0.316	0.852	0.456
91 samples	GF+MSC	13	0.939	0.291	0.877	0.417

**Figure 4:**
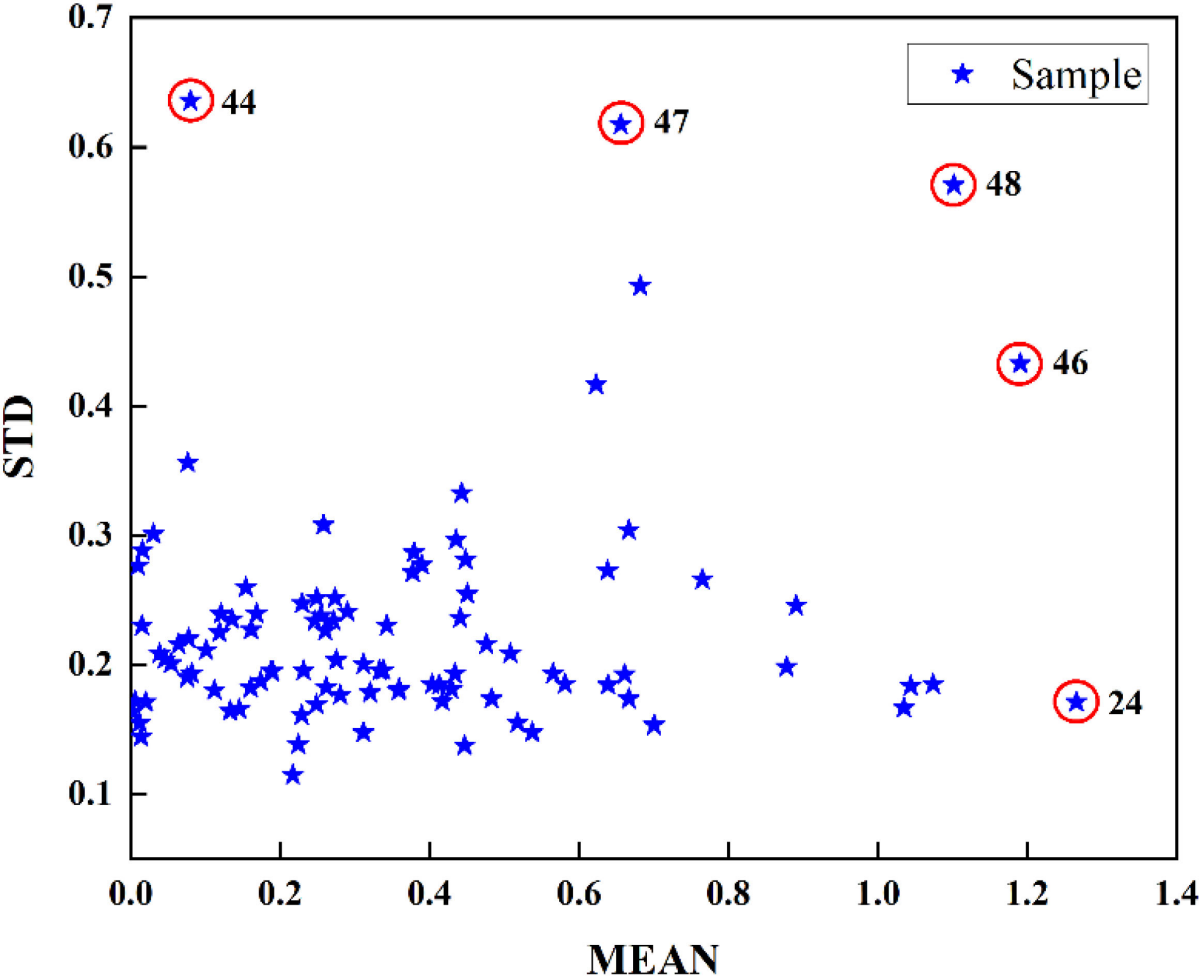
Utilization of the Monte Carlo method for the identification of outliers.

In addition, the presence of outliers within the dataset can significantly impact the characteristics of the normal samples, which leads to the inaccuracy of the established model. Before further modeling, the relevant algorithm should be used to remove the outliers from the original sample (Lepot et al., 2017; Song et al., 2022). A Monte Carlo outlier detection approach was employed to identify potential outliers within the samples. This method leverages the mean and standard deviation (STD) of the prediction error, facilitating the detection and exclusion of outliers from both spectral data and SSC (Zhang et al., 2020). As shown in **Figure 4**, samples 24, 44, 46, 47, 48 were identified as potential outliers. To validate the accuracy of the algorithm, a PLS model was developed for SSC prediction of tomato samples based on the dataset before and after removal of each potential outlier. The results were shown in **Table 2**. It can be seen that the prediction accuracies of the correction set and prediction set had been further improved ( $R_{c}\;$ = 0.939, *RMSEC* = 0.291%, $R_{p}$ = 0.877, *RMSEP* = 0.417%) after outlier removal. This suggests that the elimination of sample outliers diminished the variability within the data set, thereby enhancing the outcomes of the modeling and quantitative analysis.

### 3.3 SSC prediction of tomatoes based on effective wavelengths

#### 3.3.1 Effective wavelength selection by BVS-PLS

Each spectral curve collected from tomato samples contained 2048 wavelengths, and adjacent wavelengths had similar spectral characteristics. The selection of optimal wavelengths from comprehensive spectral variables can reduce model complexity and enhance the accuracy of detecting SSC in tomatoes. Too many wavelengths will not only lead to multicollinearity, but also increase the running time of the model. Therefore, it is essential to examine the influence of wavelengths on the model.

**Figure 5:**
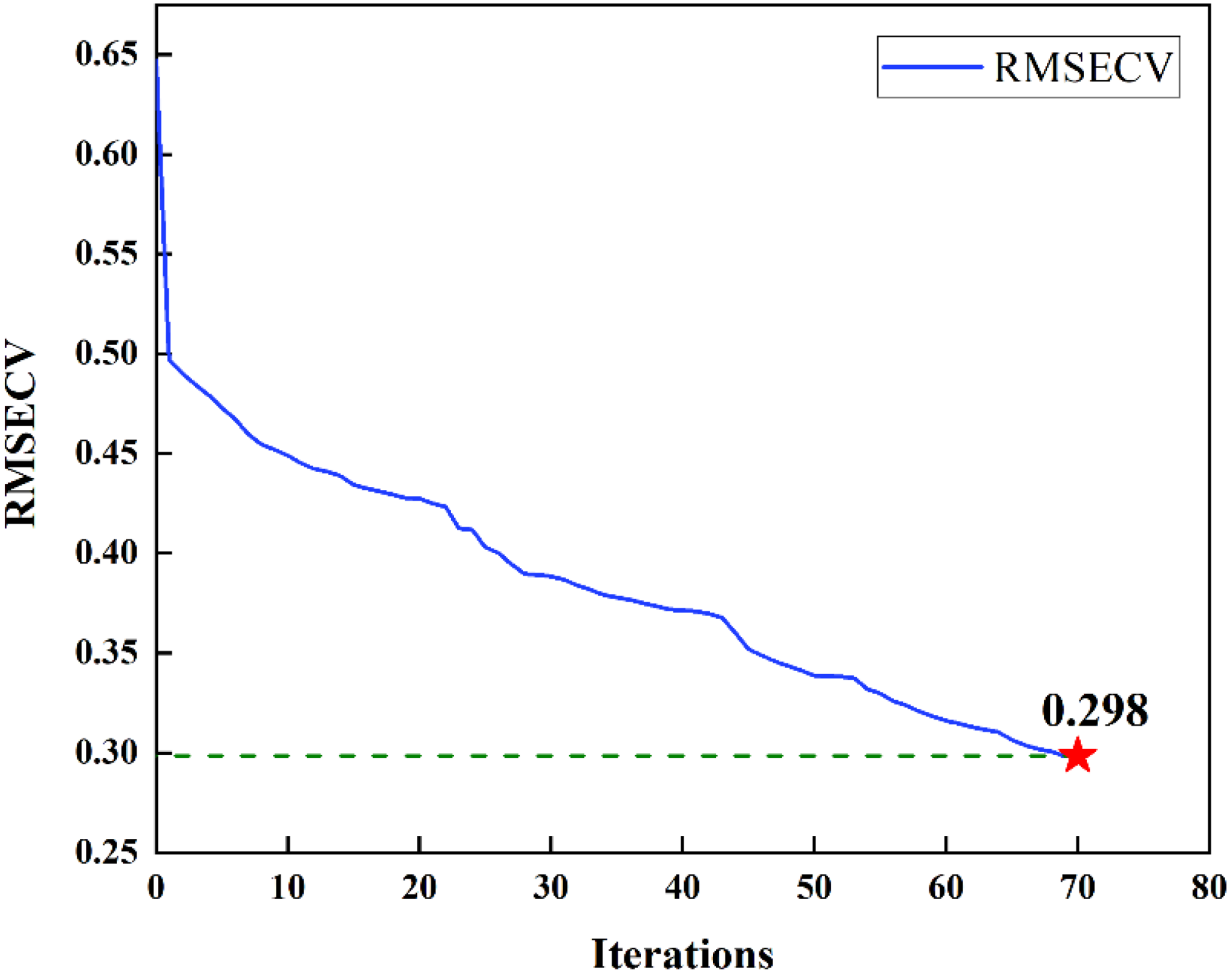
The trend of *RMSECV* values with increasing number of BVS-PLS iterations.

Too many wavelengths not only have collinearity problems, but also make the data very complicated and increase the modeling time. Since the spectra of near wavelengths reflect similar physical and chemical properties of substances, the study attempted to improve the performance of the model by dividing the full spectral bands into different groups. To examine the impact of the quantity of spectral variables on the precision of regression analysis, the spectrum containing 2048 wavelength variables was divided into several segments (n)  with the growth degree of 32, 16 and 8, respectively, and represented by the sum value of each segment, the spectrum with the number of wavelength variables (m)  of 64, 128 and 256 was obtained. The recalculated spectrum was fed into the BVS-PLS algorithm, and the variables were iteratively deleted according to the *RMSECV* value. As can be seen from **Table 3**, after several iterations, the minimum *RMSECV* values at 64, 128 and 256 spectral wavelengths were 0.469%, 0.298% and 0.415%, respectively. In general, the value of *RMSECV* decreased first and then increased, which can be attributed to the fact that the more wavelength variables, the more information of the measured object was contained in the spectrum, which was conducive to reducing the regression error; however, more wavelength variables also brought more noise, resulting in reduced accuracy. Therefore, the combined 128 wavelengths were finally used in this study to establish a tomato SSC prediction model. The iterative process of the model was shown in **Figure 5**. For each iteration, one band was deleted and 59 wavelengths were finally selected. The selected bands were used for regression analysis, and the correlation coefficients on the calibration set and prediction set were 0.955 and 0.906, respectively, and the root-mean-square errors were 0.249 and 0.369, respectively. In comparison to the full-wavelength PLS model, the predictive performance of the model enhanced following variable selection through the BVS-PLS algorithm. This suggests that the process of wavelength selection contributes positively to the optimization of the model.

**Table 3:** The minimum *RMSECV* values of BVS-PLS based on the number of different variables.

Group	Number of groups	The number of variables	*LVs*	Minimum *RMSECV (%)*
Group I	64	32	15	0.469
Group II	128	16	17	0.298
Group III	256	8	20	0.415

#### 3.3.2 Effective wavelength selection by SA algorithm

SA algorithm is a global optimization algorithm, other evolutionary methods such as genetic algorithm and particle swarm optimization may easily fall into the local optimal solution during the search process, especially in the complex high-dimensional spectral wavelength space. The SA algorithm accepts poor solutions with a certain probability, which gives it a greater chance to jump out of the local optimal and explore a wider solution space in the search process, so it is more likely to find the global optimal feature combination. In addition, the SA algorithm has better convergence. In the process of decreasing temperature, the algorithm gradually stabilizes and tends to the optimal solution. In contrast, some evolutionary methods may be deficient in convergence speed and stability, especially when dealing with large-scale spectral wavelength data. In this study, the initial temperature $T_{0}$ was set to the initial *RMSE* value and the cooling parameter α was set to 0.5% of the minimum *RMSE*. Note that the algorithm design avoided the use of a fixed value for α . This has the advantage that the cooling parameter decreases as the *RMSE* decreases, thus providing more flexibility in finding a globally optimal solution. The maximum number of iterations L was set to 500, and the termination temperature $T_{e}$ was set to a value infinitely close to zero.

The number of wavelengths selected by SA algorithm was set to 20, 30, 40 and 50 respectively, and the influence of different wavelengths on the SSC prediction accuracy of tomato was tested. As can be seen from **Table 4**, at 20 wavelengths, $R_{c}\;$ = 0.921, *RMSEC* = 0.330%, $R_{p}$ = 0.889, *RMSEP* = 0.398%; at 50 wavelengths, $R_{c}$ = 0.961, *RMSEC* = 0.232%, $R_{p}$ = 0.932, *RMSEP* = 0.306%. In general, as the quantity of wavelengths increased, the model’s accuracy on both the calibration and prediction datasets consistently enhanced, which was attributed to the fact that the more the number of spectral wavelengths, the richer the material information carried, this finding aligns with the analysis presented in section 3.3.1. However, too many wavelengths can make the prediction time of the model longer, which is not conducive to the need for online detection. Therefore, it is necessary to reduce the number of wavelengths as much as possible under the premise of ensuring the prediction accuracy of the model.

**Table 4:** Prediction results of SSC in tomatoes by PLSR model with different wavelengths selected by SA algorithm.

Number of wavelengths	*LVs*	*Rc*	*RMSEC (%)*	*Rp*	*RMSEP (%)*
20	11	0.921	0.330	0.889	0.398
30	16	0.943	0.278	0.904	0.370
40	13	0.938	0.291	0.929	0.314
50	20	0.961	0.232	0.932	0.306

#### 3.3.3 Effective wavelength selection by combination algorithm

**Figure 6:**
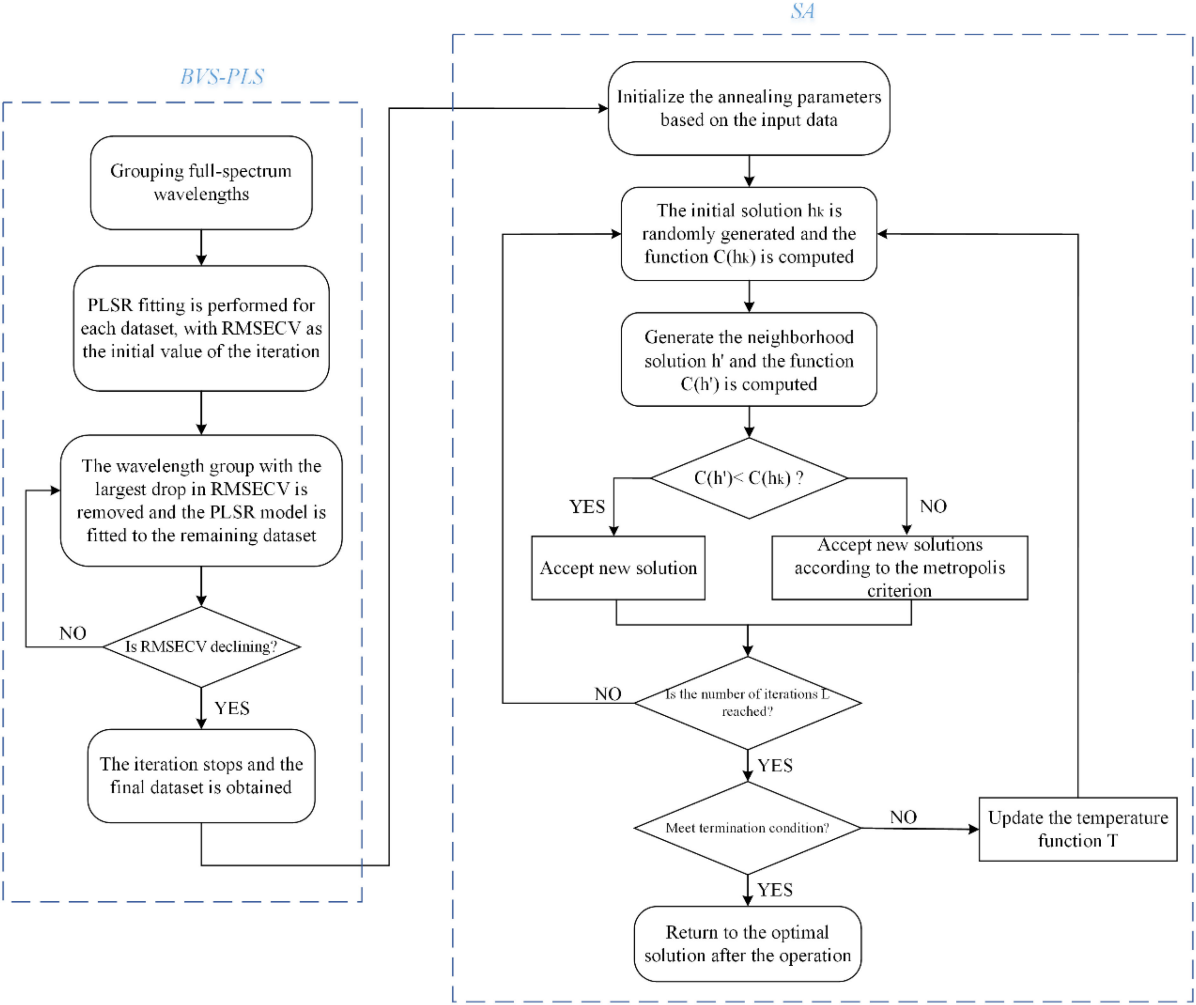
Flowchart of combinatorial feature selection algorithm.

**Figure 7:**
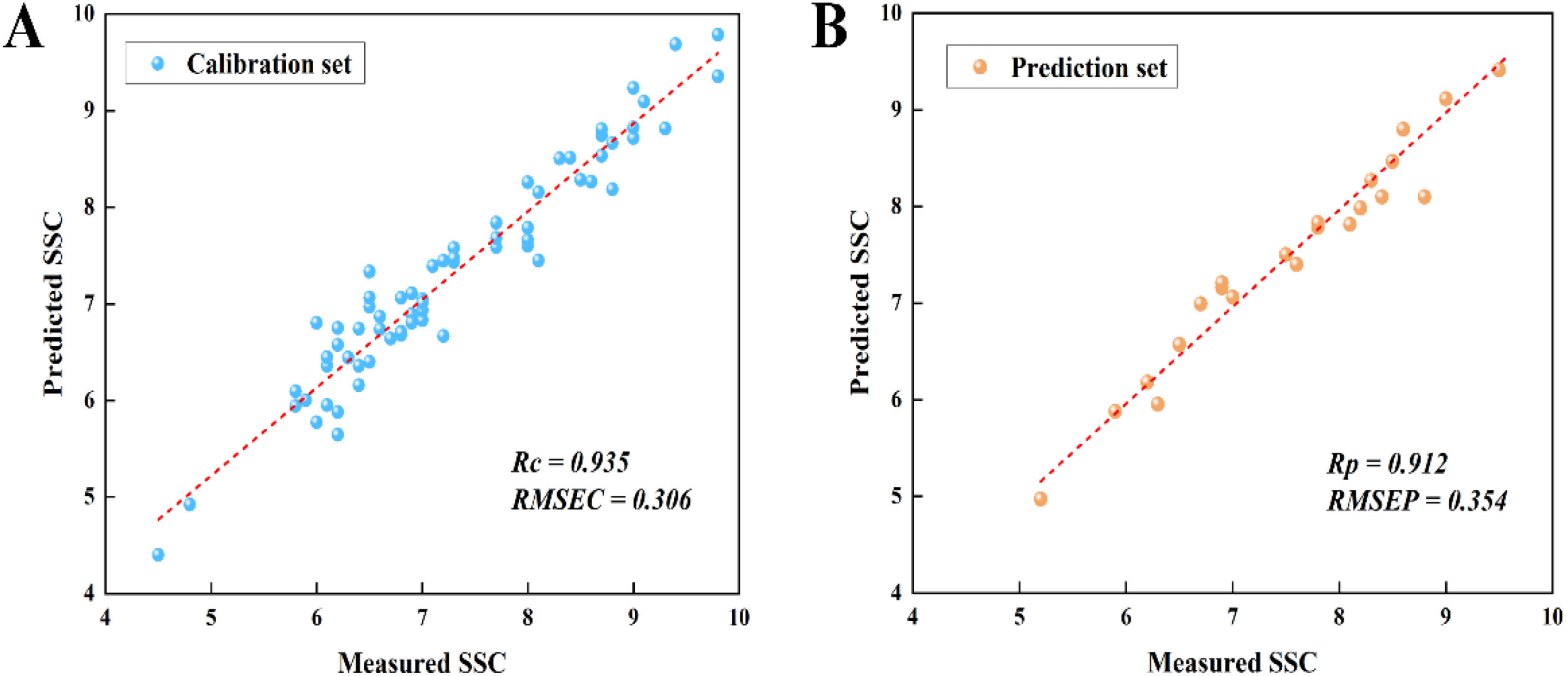
Scatter plots of predicted SSC versus measured SSC for **(A)** calibration set and **(B)** prediction set based on PLSR model constructed with wavelengths selected by combination wavelength selection strategy.

Different wavelength selection methods have their own characteristics, which can be used by combination way to improve the effect of feature selection. At the same time, the synergistic effect of multiple methods can more comprehensively assess the importance of features, so as to screen out more representative spectral wavelengths (Gerretzen et al., 2015; Zhao et al., 2018; Shen et al., 2020). In this study, BVS-PLS algorithm was first used to select the spectral wavelengths and eliminate the non-information variables, and then SA algorithm was used to further reduce the multicollinearity between the variables (**Figure 6**). In order to evaluate the effectiveness of the algorithm, based on 59 wavelengths selected by BVS-PLS algorithm, the parameter settings were kept unchanged, and 20 spectral wavelengths were further selected by SA algorithm. The PLSR model was constructed based on the selected final wavelengths, and the model prediction results were shown in **Figure 7**. $R_{c}\;$ = 0.935, *RMSEC* = 0.306%, $R_{p}$ = 0.912, *RMSEP* = 0.354%, compared with the model constructed using only the wavelength selected by BVS-PLS algorithm, the accuracy of the prediction set was further improved. At the same time, compared with the PLSR prediction model constructed with 20 wavelengths selected by SA algorithm only, the wavelength selected by dual feature selection algorithm had better prediction performance for tomato SSC, and compared with the PLSR model constructed with 50 wavelengths selected by SA algorithm only, the prediction accuracy obtained by using dual feature selection algorithm only decreased a little, however, the number of wavelengths was dramatically reduced. In general, compared with 2048 wavelengths in the full-spectrum model, after grouping spectral wavelengths, BVS-PLS and SA screening, the PLSR model established using only 20 selected wavelengths (only 0.97% of the original spectrum) showed acceptable and robust prediction ability. This meant that the proposed wavelength selection technique can significantly simplify and improve the model performance, which was of great help to meet the demand of fast detection in the actual production line.

## 4 Conclusions

In this study, SSC of tomato was successfully measured under different acquisition orientation based on the developed multi-point Vis-NIR full transmission spectrum acquisition system. Four methods, MSC, SNV, GF and GF and MSC, were used to pretreat the original transmission spectrum of the tomato samples, and the Monte Carlo outlier detection method was employed to identify anomalous samples, while the effective wavelengths were determined using BVS-PLS, SA, and a combination of BVS-PLS and SA, respectively. Finally, PLSR linear model was established to predict tomato SSC. The results showed that the prediction performance of Z2 orientation was better than that of Z1 orientation, a shorter optical propagation path can build more stable model. Therefore, in the actual measurement, the orientation of the tomato should be placed according to Z2, so as to obtain the high-quality spectral data through the spectrometer. After abnormal sample removal and GF and MSC treatment, the spectral curve was smoother and the spectral scattering effect was suppressed. The feature selection algorithm combined with BVS-PLS and SA selected 20 effective spectral wavelengths from the original 2048 variables, and the prediction accuracy of $\;R_{p}$ = 0.912 and *RMSEP* = 0.354% was obtained. In summary, the variable screening algorithm developed in this study can greatly reduce irrelevant information variables in the original spectral data while ensuring the accuracy of the model, thus eliminated multicollinearity in the spectral wavelengths and greatly improved the operating efficiency of the model. Next step of work, more tomato varieties and samples were used to improve and optimize the model performance for online application.

## Data Availability

The original contributions presented in the study are included in the article/supplementary material. Further inquiries can be directed to the corresponding author.
